# Effect of Extrusion on Mechanical Property, Corrosion Behavior, and In Vitro Biocompatibility of the As-Cast Mg-Zn-Y-Sr Alloy

**DOI:** 10.3390/ma17061297

**Published:** 2024-03-11

**Authors:** Tao Huang, Lijing Yang, Chunxiang Xu, Cheng Xu, Qingke Zhang, Jinshan Zhang, Zhenlun Song

**Affiliations:** 1 Key Laboratory of Marine Materials and Related Technologies, Zhejiang Key Laboratory of Marine Materials and Protective Technologies, Ningbo Institute of Materials Technology and Engineering, Chinese Academy of Sciences, Ningbo 315201, China; 2 College of Materials Science and Engineering, Taiyuan University of Technology, No. 79 Yingze West Street, Taiyuan 030024, China

**Keywords:** Mg, extrusion, grain refinement, galvanic corrosion, MTT

## Abstract

The effect of extrusion on the microstructure, mechanical property, corrosion behavior, and in vitro biocompatibility of as-cast Mg-1.5Zn-1.2Y-0.1Sr (wt.%) alloy was investigated via tensile tests, electrochemical methods, immersion tests, methylthiazolyl diphenyltetrazolium bromide (MTT), and analytical techniques. Results showed that the as-cast and as-extruded Mg-1.5Zn-1.2Y-0.1Sr alloys comprised an α-Mg matrix and Mg_3_Y_2_Zn_3_ phase (W-phase). In the as-cast alloy, the W-phase was mainly distributed at the grain boundaries, with a small amount of W-phase in the grains. After hot extrusion, the W-phase was broken down into small particles that were dispersed in the alloy, and the grains were refined considerably. The as-extruded alloy exhibited appropriate mechanical properties that were attributed to refinement strengthening, dispersion strengthening, dislocation strengthening, and precipitation strengthening. The as-cast and as-extruded alloys exhibited galvanic corrosion between the W-phase and α-Mg matrix as the main corrosion mechanism. The coarse W-phase directly caused the poor corrosion resistance of the as-cast alloy. The as-extruded alloy obtained via hydrogen evolution and mass loss had corrosion rates of less than 0.5 mm/year. MTT, high-content screening (HCS) analysis, and cell adhesion tests revealed that the as-extruded alloy can improve L929 cell viability and has great potential in the field of biomedical biodegradable implant materials.

## 1. Introduction

Metals, polymers, composites, and ceramics are the most common materials used for biomedical implants [1,2]. Among these, Magnesium (Mg) metal has received increasing attention given its degradability, excellent biocompatibility, and mechanical compatibility (e.g., elastic modulus and density close to those of the human bone) [3,4,5]. However, insufficient mechanical properties [6] and a fast corrosion rate [7] (especially in electrolytes containing chloride ions [8]) limit its clinical application. Erinc et al. [9] put forward criteria to evaluate the Mg used in biodegradable implants: (1) corrosion rate in simulated body fluid (SBF) at 37 °C < 0.5 mm/year; (2) yield strength (YS) > 200 MPa and elongation (EL) > 15%. Thus, the mechanical properties and corrosion resistance of Mg material must be further improved to meet practical needs.

The performance of Mg can be ameliorated through various means, one of which is alloying [10,11]. Alloying elements should be nontoxic or have extremely low toxicity. Based on this principle, Zn is widely used in Mg materials. Numerous studies [12,13,14,15] have found that Zn can simultaneously improve mechanical properties and corrosion resistance. Hence, Mg-Zn alloys have attracted great interest from many researchers. In addition, Y is often used with Zn to improve the performance of Mg because the former can form a Mg-Y-Zn ternary phase with Mg and Zn, which has a notable strengthening effect on mechanical properties. Jiang et al. [16] reported that the Mg-5.1Zn-3.2Y-0.4Zr-0.4Ca alloy containing the W-phase had a high strength with YS and an ultimate tensile strength (UTS) exceeding 330 MPa and 350 Mpa, respectively. Y also plays a role in the improvement of corrosion resistance. Panemangalore et al. [17] found that Y decreased the current density of Mg-5Sn alloys, and the presence of Y in the oxide layer made the substrate less susceptible to corrosion. Sr has received increasing attention due to its excellent modification effect. Cheng et al. [18] reported that Sr can refine the grain size, and it showed an evident improvement on the mechanical properties and corrosion resistance of the Mg-5Zn alloy. Kiani et al. [19] also arrived at a similar conclusion. Moreover, other reports showed that Sr can promote osteocyte differentiation and prevent thrombosis [20,21]. Hence, Mg-Zn-Y-Sr is an alloy with promising biomedical applications, but it has rarely been studied. Based on this background, in this work, a relatively excellent alloy with Mg-1.5Zn-1.2Y-0.1Sr (wt.%) composition was prepared via casting according to previous research.

Unfortunately, it is still difficult for the as-cast Mg-1.5Zn-1.2Y-0.1Sr alloy to meet the clinical application requirements. Other methods, such as hot extrusion, should be adopted to further enhance the performance of Mg alloys. Hot extrusion can refine the microstructure and optimize the distribution of the second phase, which has a significant impact on property. As reported by Gui et al. [22], the UTS, YS, and EL of the as-extruded Mg-1.59Nd-2.91Zn-0.05Zr-0.35Mn alloy increased by 71.6%, 109.9%, and 143.7%, respectively, compared with those of the as-cast alloy. The excellent properties of the as-extruded alloy were attributed to a special microstructure comprised of multiscale a-Mg grains and precipitates, which provide grain boundary strengthening, solution strengthening, and precipitation strengthening. Shi et al. [23] revealed that fine grain strengthening and dispersion strengthening after hot extrusion were the main reasons for the improvement of the mechanical properties of the Mg-4Zn-0.5Sr-0.5Ag alloy. In addition, the second phase of fine dispersion in the as-extruded Mg-4Zn-0.5Sr-0.5Ag alloy was conducive to the formation of “small cathode–large anode” electric couples, which considerably reduced the corrosion rate. Jafari et al. [24] also found that extrusion process can simultaneously improve corrosion resistance and mechanical properties, and especially corrosion resistance. The corrosion rate of the Mg-5Zn-1.5Y alloy was reduced by 80% due to sufficient dynamic recrystallization and the decreased area percentage of intermetallics after extrusion at high temperature (460 °C).

In summary, the role of extrusion provides an idea for improving the properties of Mg alloys. However, very limited investigations have been focused on the influence of extrusion on the properties of as-cast Mg-Zn-Y-Sr alloys. Hence, we conducted hot extrusion on the as-cast Mg-1.5Zn-1.2Y-0.1Sr alloy. Through a study of the differences in microstructure, mechanical property, corrosion behavior, and in vitro biocompatibility between the as-cast and as-extruded alloys, the specific effects of hot extrusion on the as-cast alloy were discussed in detail. The feasibility of the Mg-1.5Zn-1.2Y-0.1Sr alloy used as biological implant material was verified in this paper.

## 2. Materials and Methods

### 2.1. Materials

Mg-1.5Zn-1.2Y-0.1Sr (wt.%) alloys were prepared using high-purity Mg (≥ 99.99 wt.%), Zn (≥ 99.99 wt.%), Y (≥ 99.99 wt.%), and Mg-20 wt.% Sr master alloy via the traditional gravity casting method. The raw materials were melted at 730 °C for 30 min under a mixed protective atmosphere (99 vol% CO_2_ and 1 vol% SF_6_), and then poured into a mild steel mold (200 ± 5 °C) to obtain an alloy ingot. The element composition of the as-cast alloy was measured using an inductively coupled plasma optical emission spectrometer (ICP-OES, SPECTRO ARCOS, SPECTRO, Kleve, Germany) (Table 1). The Zn, Y, and Sr contents coincided with the designed values, and the deviations were in an acceptable range. Other trace elements were comparable with the high-purity Mg. After casting, the ingot was heated to 480 °C for 24 h and quenched with water at 80 °C. The solution-treated alloys were machined into cylinders with a dimension of Φ 40 mm × 40 mm and pre-heated at 330 °C for 2 h. Then, a YAW-5000 vertical extruder (Dongchen corporation, Jinan, China) was used to extrude the solution-treated alloy in a rod at an extrusion ratio of 1:16. During the extrusion process, the extrusion rate and temperature were maintained at 60 mm/min and 300 °C, respectively [25]. Finally, extrusion bars cooled at room temperature with a radius of 5 mm were obtained.

### 2.2. Microstructural Characterization and Mechanical Tests

The specimens were ground to 2000#, polished, and then etched with 4% nital solution (as-cast) or picral etching reagent (as-extruded) consisting of 1.5 g picric acid, 5 mL acetic acid, 25 mL ethanol, and 5 mL deionized water. An optical microscope (OM, NMM-800RF, Yongxin Ltd., Ningbo, China) instrument and a scanning electron microscope (SEM, Sirion200, FEI, Hillsboro, OR, USA) equipped with an energy dispersive X-ray spectroscope (EDS) were utilized to characterize the microstructure and analyze the elemental composition. Phase composition identification was carried out using X-ray diffraction (XRD, D8 ADVANCE, Bruker, Karlsruhe, Germany) equipment with Cu-Kα radiation, and the scanning angles were from 10° to 90° with a speed of 6°/min. Phase structure characterization was performed with a transmission electron microscope (TEM, Tecnai F20, FEI, Hillsboro, OR, USA) operating at 200 kV. The samples for TEM observation were ground to 50 μm, and then ion-beam-polished by a Gatan695 device until the sample center was perforated. The thermal analysis of the as-cast alloy was conducted via differential scanning calorimetry (DSC, STA449F3, Netzsch corporation, Selb, Germany) at a heating rate of 10 °C/min under 1 bar argon flow with a purge rate of 0.1 L/min.

### 2.3. Mechanical Tests

The specimens used for the tensile tests had a thickness of 2 mm, a gauge length of 18 mm, and a width of 4 mm. The tensile tests were carried out at room temperature with a DNS100 material-testing machine (CIMACH, Changchun, China) at a constant speed of 0.2 mm/min. At least three specimens were prepared to guarantee the repeatability. The fracture morphology was obtained by SEM. Microhardness (Vicker hardness, HV_0.1_) was tested seven times for each alloy on an HV 1000 instrument (SIOMM, Shanghai, China) with a load of 100 g and holding time of 15 s.

### 2.4. Electrochemical Measurements

The electromechanical tests involved a standard three-electrode configuration with a saturated calomel electrode (SCE) as the reference electrode, a platinum sheet (10 mm × 10 mm) as the counter electrode, and the sample as the working electrode. Samples used for electromechanical tests were sealed with epoxy resin with an exposed area of about 0.75 cm^2^, and the tested surface was the transverse plane. Before the experiments, samples were ground to 2000#, polished, cleaned in acetone (10 min), and dried in warm air. Specimens were immersed in SBF [26] for 30 min at 37 ± 0.5 °C to reach a relatively stable open-circuit potential (OCP). Then, electrochemical impedance spectroscopy (EIS) was performed at the frequency range from 10^5^ Hz to 10^−2^ Hz with an amplitude of 5 mV. A potentiodynamic polarization (PDP) test was conducted at a scanning rate of 1 mV/s in a potential range of ± 500 mV. The EIS data and polarization curves were analyzed using ZView3.1 and Nova1.11 software, respectively.

### 2.5. Immersion Tests

Hydrogen evolution and mass loss tests were performed in SBF at 37 ± 0.5 °C to evaluate the corrosion rates. The SBF temperature was maintained using a water bath. The sample was fixed utilizing a fine nylon rope through the drilled hole, and the amount of hydrogen produced was collected using an inverted funnel. A hydrogen evolution device can be seen as described by Song [27]. The ratio of the sample surface area to the SBF volume was 1 cm^2^: 40 mL. Before the experiments, the specimens were ground to 2000#, polished, cleaned, blow-dried, and weighed (m_1_). The pH of the electrolyte was monitored after immersion at different intervals. After 240 h immersion, the concentration of Mg ions in the SBF was measured by ICP-OES. Meanwhile, the corrosion products were analyzed via XRD, SEM, and EDS, Fourier transform infrared spectroscopy (FTIR, 6700, NICOLET, Madison, WI, USA), and X-ray photoelectron spectroscopy (XPS, AXIS SUPRA, KRATOS, Manchester, UK). Then, the corrosion layer formed on the surface of samples was removed using chromic acid in a ratio of 200 g chromium trioxide to 1 L deionized water and weighed again (m_2_). The corrosion rate (CR) after 240 h immersion can be calculated according to ASTM G1-03 using the following formula:

(1) $$\mathrm{CR}=\frac{{8.76\times 10}^{4}\times (m_{1}\;-\;m_{2})}{S\times T\times \rho }$$

where m_1_ − m_2_ (g) is the reduction in sample mass obtained from mass loss and hydrogen evolution (1 mL H_2_ = 0.001083 g Mg [28]), S refers to the surface area of samples (cm^2^), T indicates the immersion time (h), and ρ represents the density of alloys (1.74 g/cm^3^). At least three specimens were prepared to guarantee the repeatability of experiment results.

### 2.6. Cytotoxicity Tests

Murine fibroblast cells (L929, provided by the Cell Bank of the Chinese Academy of Sciences, Shanghai, China) were adopted to evaluate the cytotoxicity of the as-cast and as-extruded alloys via MTT assay according to ISO 10993-5:2009 [29]. The cells were cultured in Dulbecco’s modified Eagle’s medium (DMEM) supplemented with double-antibody (80 μg/mL streptomycin and 80 U/mL penicillin) and 10% fetal bovine serum at 37 °C, 95% relative humidity, and 5% CO_2_. Prior to the tests, specimens were sterilized with 75% alcohol solution for 30 min and ultraviolet radiation for 120 min. Extracts were prepared at a DMEM volume to sample surface area ratio of 1.25 mL/cm^2^ in an incubator at 37 °C for 72 h. The pH and concentrations of Mg, Zn, Y, and Sr ions in the extracts were measured. The supernatant fluid was collected, filtered, and diluted (100%, 50%, and 25%) with fresh DMEM. L929 cells were seeded on a 96-well flat-bottomed culture plate (5 × 10^3^ cells/well) and cultured for 24 h to allow cell attachment. Then, the medium was discarded and replaced with 100 μL DMEM (negative control) and extracts. After 24 h, 48 h, and 72 h incubation, 10 μL MTT at 5 mg/mL concentration was added to each well and reacted for 4 h. Finally, the MTT-containing extracts were replaced with 150 μL dimethyl sulfoxide and shaken for 10min. Absorbance was recorded using SpectraMax190 microplate reader (Molecular Devices, San Francisco, CA, USA) at 550 nm wavelength.

### 2.7. Cell Morphology

The L929 cells (5 × 10^3^ cells/well) incubated in the 96-well plate with 100% extracts for 24 h, 48 h, and 72 h were analyzed after staining with fluorescein isothiocyanate (FITC) for the cytoskeleton and 4′,6-diamidino-2-phenylindole (DAPI) for the nuclei. Firstly, 4% paraformaldehyde was added to each well to fix the cells. After 20 min, paraformaldehyde was discarded, and the culture plate was washed thrice with phosphate-buffered saline (PBS). Then, the cells were stained with FITC for 30 min and DAPI for 20 min, successively. Finally, the images of nuclei (blue) and cytoplasm (green) were obtained using the Operetta CLS (PerkinElmer, Waltham, MA, USA).

### 2.8. Cell Adhesion

The Mg alloy sample discs, L929 cells, and 24-well plates were used to investigate cell adhesion behavior. In total, 100 μL L929 cell suspension in DMEM (around 1 × 10^4^ cells) was seeded on the sample surface and cultured at 37 °C with 5% CO_2_ for 1 h to allow cell attachment, after which 1 mL DMEM was added to each well and further cultured at 37 °C for 24 h. Then, the samples were fixed with 4% paraformaldehyde for 2 h and rinsed thrice with PBS, followed by dehydration in 25 vol%, 50 vol%, 75 vol%, 90 vol%, and 100 vol% ethanol/distilled water gradient, successively. After drying in a 37 °C oven for 24 h, the cell morphology was observed using an 8230 SEM.

### 2.9. Statistical Analysis

The results were expressed as the means ± standard deviation. All data were statistically analyzed by variance analysis, and *p*-values less than 0.05 were considered to be statistically significant.

## 3. Results

### 3.1. Microstructural Analysis

**Figure 1:**
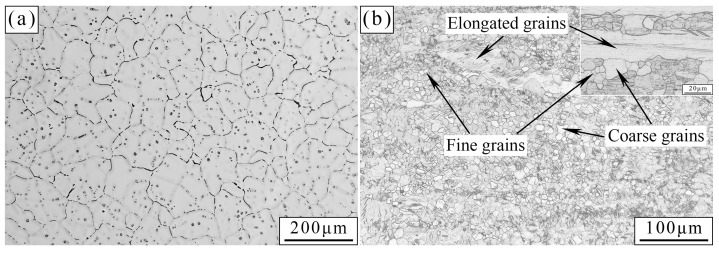
OM images of the (**a**) as-cast and (**b**) as-extruded alloys. The inset in (**b**) shows the longitudinal section of the as-extruded alloy.

Figure 1 shows the OM images of the as-cast and as-extruded alloys. The as-cast alloy presents an irregular petal shape, and dendrite structures are evident. A majority of grain sizes are in the range of 50–200 μm. After hot extrusion, the grains are refined considerably. The grains of the as-extruded alloy can be classified as coarse, fine, and elongated. The coarse and fine grains have sizes of 5–20 μm and 1–5 μm, respectively. The coarse and fine grains were the result of dynamic recrystallization [30,31]. During hot extrusion, a high extrusion temperature creates dynamic conditions and a high extrusion ratio provides nucleation sites for dynamic recrystallization [32]. The size of elongated grains which were suggested to have survived the dynamic recrystallization is over 100 μm. The mixed grain feature can be attributed to the energy inhomogeneity of the alloy during extrusion. When the plastic stored energy in a certain region is inadequate to start dynamic recrystallization, the grain will be elongated [33].

**Figure 2:**
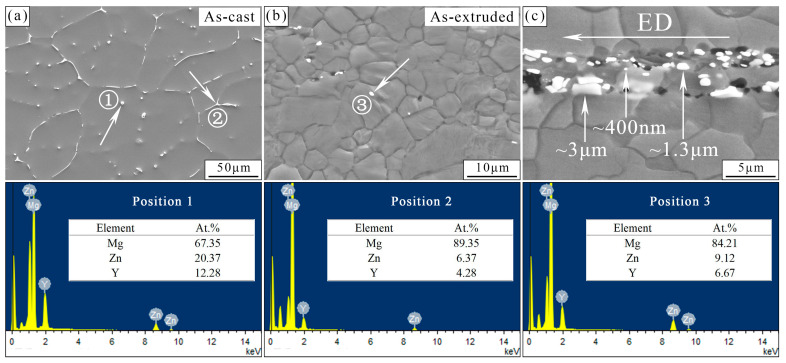
Figure (**a**) and (**b**) represent the SEM images of the as-cast and as-extruded, respectively. Figure (**c**) shows the longitudinal section of the as-extruded alloy.

Figure 2 presents the SEM images of the as-cast and as-extruded alloys. The eutectic compounds in the as-cast alloy are mainly distributed semi-continuously at the grain boundaries, and some granular precipitates with a size of 1–5 μm appear in the grains. After hot extrusion, the eutectic compounds are broken into small particles and dispersed along the extrusion direction (ED). The small particles have a size of 1–3 μm (micron-sized) and 0–1 μm (nano-sized). The micron-/nano-sized precipitates and coarse, fine, and elongated grains form a multiscale structure. EDS results show that the eutectic compounds in the as-cast and as-extruded alloys may be Mg_3_Y_2_Zn_3_ due to a Zn/Y ratio close to 3/2. Sr was too low to be detected.

**Figure 3:**
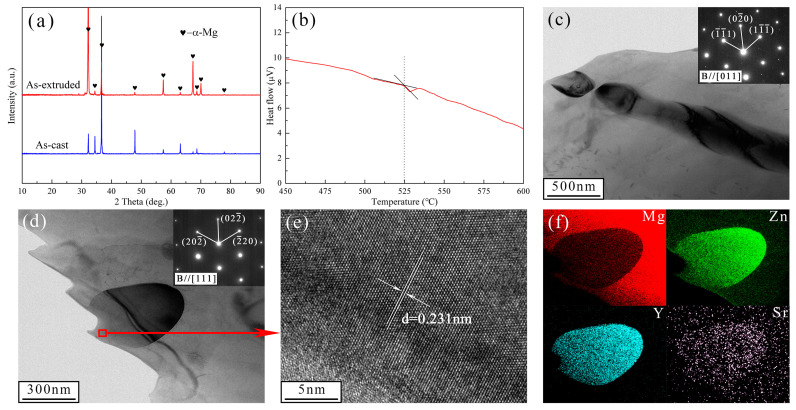
(**a**) XRD patterns, (**b**) DSC curve of the as-cast alloy, TEM images of the (**c**) as-cast and (**d**) as-extruded alloys, (**e**) HRTEM of W-phase in the as-extruded alloy, (**f**) EDS mapping of the as-extruded alloy. The insets in (**c**,**d**) represent the SAED patterns.

Figure 3a shows the XRD patterns of the as-cast and as-extruded alloys. Except for the α-Mg matrix, no other phases were detected due to the low content of alloying elements. To confirm the species of the second phase, we executed a thermal analysis of the as-cast alloy via DSC (Figure 3b). Results show that the as-cast Mg-1.5Zn-1.2Y-0.1Sr alloy had an endothermic peak at approximately 525 °C, which indicates that the melting temperature of the second phase is around 525 °C. As reported in Ref. [34], the phase transition temperature of the W-phase was 521 °C (L → α-Mg + Mg_3_Y_2_Zn_3_). In this work, the phase transition temperature differed from that of Mg-Zn-Y ternary phase diagram, possibly due to lattice distortions or local lattice distortions, which reduced the binding energy between atoms. Thus, the second phase in the Mg-1.5Zn-1.2Y-0.1Sr alloy was the W-phase. Figure 3c,d present the TEM images of the as-cast and as-extruded alloys, respectively. Selected area electron diffraction (SAED) patterns (the insets in Figure 3c,d) confirm that the W-phase has an fcc structure. The W-phase in the as-cast alloy shows a lattice parameter with *a* of 0.6866 nm, and the W-phase in the as-extruded alloy exhibits a lattice parameter with *a* of 0.6918 nm, which accords with the report on the W-phase structure in the Mg-5.18Zn-1.29Y-0.98Zr alloy [35]. The high-resolution transmission electron microscopy (HRTEM) analysis shows lattice fringes with a spacing of 0.231 nm, which correspond to the (220) crystal face of the W-phase. EDS mapping (Figure 3f) demonstrates the uniform distribution of Mg in the α-Mg matrix, and the concentrations of Zn, Y, and Sr in the W-phase.

### 3.2. Mechanical Properties

**Figure 4:**
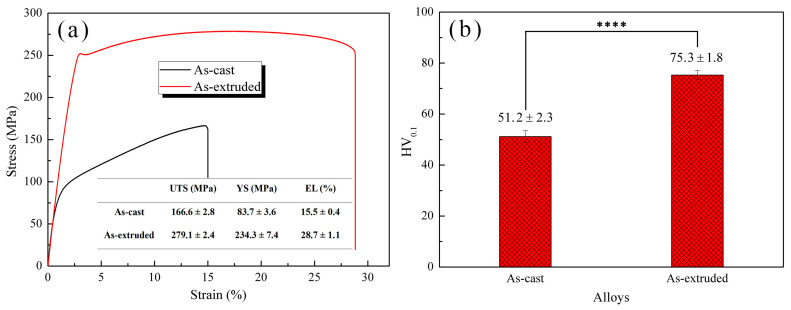
(**a**) Stress–strain curves and (**b**) Vickers hardness of the as-cast and as-extruded alloys. **** *p* < 0.0001.

Figure 4a presents the stress–strain curves of the as-cast and as-extruded alloys. The as-cast alloy has low tensile properties, with UTS, YS, and EL of 166.6 ± 2.8 MPa, 83.7 ± 3.6 MPa, and 15.5% ± 0.4%, respectively. After hot extrusion, the tensile properties improved considerably, with UTS, YS, and EL increasing by around 68%, 180%, and 85%, respectively. The Vickers hardness also increased by about 45% (Figure 4b).

**Figure 5:**
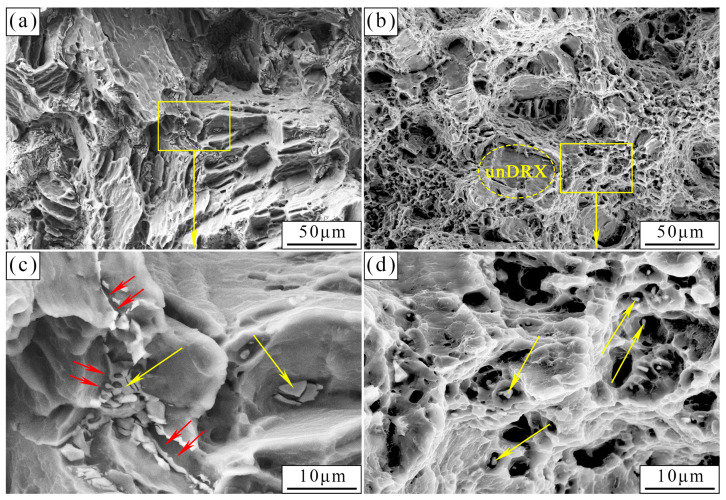
Fracture morphology of the (**a**,**c**) as-cast and (**b**,**d**) as-extruded alloys. Red arrows in (**c**) represent the microcracks at the grain boundaries. Yellow arrows in (**c**,**d**) represents the retained W-phase.

Figure 5 depicts the fracture morphology of the as-cast and as-extruded alloys. Evidently, the as-cast alloy suffered from transgranular cleavage rupture. Plenty of smooth cleavage planes and torn edges were observed, along with a few dimples. The coarse W-phases distributed at the grain boundaries and within the grains were retained (marked by yellow arrows), and microcracks at the grain boundaries were observed (marked by red arrows). For the as-extruded alloy, the fracture mode is dominated by microvoid coalescence fractures, accompanied by the formation of a large number of dimples. Numerous small W-phases are left at the bottom of the dimple, which is a prominent feature of microvoid coalescence fracture; this observation will be discussed later. A small number of cleavage planes (marked by an ellipse in Figure 5b) originate from the grains that skipped dynamic recrystallization (unDRX).

### 3.3. Degradation Behavior

**Figure 6:**
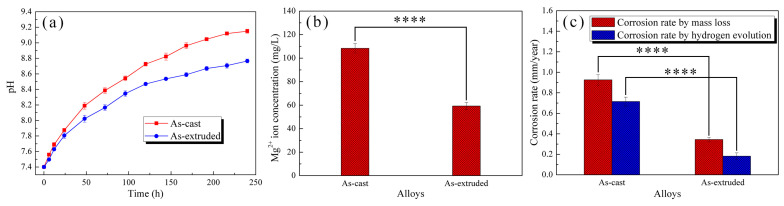
(**a**) pH variations during 240 h immersion in SBF, (**b**) Mg^2+^ ion concentration, and (**c**) corrosion rates after immersion in SBF for 240 h. **** *p* < 0.0001.

Figure 6a shows the pH variations of the as-cast and as-extruded alloys during 240 h immersion in SBF. The pH rises rapidly in the early stage, and then slows down gradually. The pH of the as-cast alloy increases faster than of the as-extruded alloy, with the latter showing a smaller value, which indicates that the as-extruded alloy has a better corrosion resistance, which can be reflected by the Mg^2+^ release concentration (Figure 6b). After 240 h immersion, the corrosion rates of the as-cast alloy calculated via hydrogen evolution and mass loss were 0.71 ± 0.04 mm/year and 0.93 ± 0.05 mm/year, respectively, and those of the as-extruded alloy were 0.18 ± 0.03 mm/year and 0.34 ± 0.02 mm/year, respectively. The corrosion rate calculated via hydrogen evolution was lower than that calculated using weight loss, and such a result is ascribed to the dissolution of hydrogen gas.

**Figure 7:**
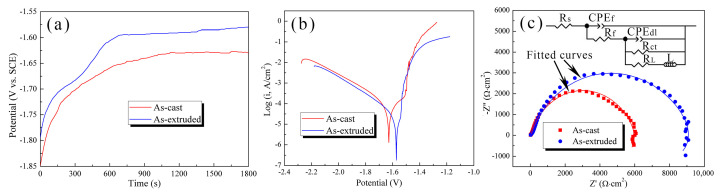
Curves of (**a**) OCP, (**b**) PDP, and (**c**) Nyquist plot measured in SBF. The inset in (**c**) represents equivalent circuits.

Figure 7a shows the OCP curves of the as-cast and as-extruded alloys immersed in SBF for 30 min. The OCP rises rapidly at first and then stabilizes. The increase in OCP (0–600 s) implies the initiation and propagation of corrosion, and the relatively stable OCP (600–1800 s) means that the corrosion of the substrate and deposition of corrosion products reach a dynamic balance. The higher the OCP, the less susceptible the alloy is to corrosion. The as-extruded alloy has a higher stable OCP than the as-cast alloy, which indicates the better corrosion resistance of the former.

Figure 7b presents the PDP curves of the as-cast and as-extruded alloys measured in SBF. The anodic curves represent the Mg oxidation process, and the cathodic curves represent the reduction reaction that produces hydrogen. Table 2 provides a summary of the corrosion potential (E_corr_), corrosion current density (J_corr_), and pitting potential (E_pt_). The J_corr_ was obtained using cathodic extrapolation due to the negative difference effect (NDE) [36]. J_corr_ can accurately reflect the magnitude of the corrosion rate, and the corrosion rate decreases with the decrease in J_corr_ [37]. The as-extruded alloy has lower J_corr_ than the as-cast alloy, which implies the better corrosion resistance of the former. The E_corr_ reflects the corrosion driving force, and a positive E_corr_ indicates low corrosion tendency [38]. After extrusion, the dispersive W-phase renders the positive shift in E_corr_ [39]. Hence, the as-extruded alloy is less susceptible to corrosion. The presence of E_pt_ indicates the breakdown of passive film due to localized corrosion, and a negative E_pt_ means a likely localized corrosion [40]. The as-cast alloy attains a higher E_pt_ than the as-extruded alloy, which means that the as-extruded alloy is more prone to pitting corrosion.

Figure 7c depicts the Nyquist plots obtained from EIS measurements. The curve shapes of the as-cast and as-extruded alloys are similar, which indicates their similar corrosion mechanism. The Nyquist plots reveal two capacitive loops and one inductive loop. The capacitive loop at high frequency is attributed to the formation of corrosion film on the alloy surface, and that at middle frequency is ascribed to the charge transfer process of Mg/Mg^2+^ at the double layer [41]. The diameter of the middle-frequency capacitive loop is near to the charge transfer resistance of the working electrode [42]. Hence, the as-extruded alloy has a better corrosion resistance than the as-cast alloy because of the larger diameter of the former. The inductive loop at low frequency is related to the relaxation of corrosion products due to the adsorption of Mg^+^ intermediates [43]. Meanwhile, the presence of a low-frequency inductive loop indicates local corrosion induced by Cl^−^ [30]. To further understand the corrosion characteristics of the as-cast and as-extruded alloy, we built an equivalent circuit model (the inset in Figure 7c). The fitting data are presented in Table 3. R_s_ represents the solution resistance. CPE_f_ and R_f_ are the constant phase element of the corrosion film and film resistance, respectively. CPE_f_ is related to the corrosion film thickness, and a low CPE_f_ indicates a thick film [44]. Severe corrosion results in the formation of a thick corrosion film on the as-cast alloy surface. Nonetheless, the as-extruded alloy has a higher R_f_, which implies the better protective effect of its corrosion products on the substrate. CPE_dl_ and R_ct_ represent the constant phase element of the product film and charge transfer resistance, respectively. CPE_dl_ is related to the corrosion reaction area, and the higher the CPE_dl_, the larger the corrosion reaction area [45]. R_ct_ reflects the corrosion resistance, and a high R_ct_ indicates a low corrosion rate. CPE_dl_ and R_ct_ confirm that the corrosion resistance of the as-extruded alloy is considerably better than that of the as-cast alloy, which is consistent with the results of immersion tests. Inductance (L) and inductance resistance (R_L_) mean the presence of pitting corrosion.

**Figure 8:**
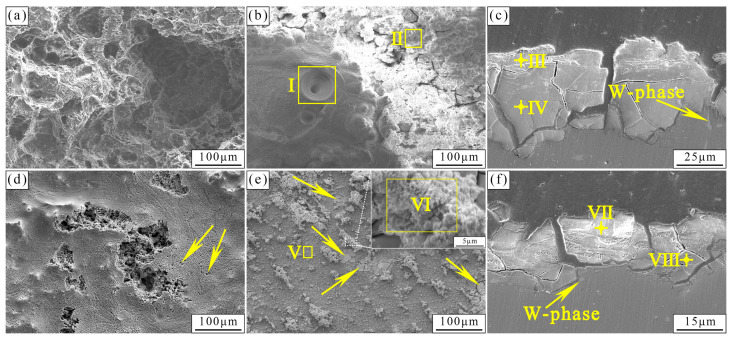
Corrosion morphologies, surface corrosion products, and longitudinal section images of the (**a**–**c**) as-cast and (**d**–**f**) as-extruded alloys after 240 h immersion. The arrows in (**d**) represent small pits left by the detachment of the W-phase. The arrows in (**e**) show a local rupture caused by pitting corrosion.

Figure 8 shows the corrosion morphology, surface corrosion products, and longitudinal section images of the alloys after 240 h immersion in SBF. Severe corrosion results in the formation of huge corrosion pits on the as-cast alloy surface. Corrosion products present a volcano-like morphology, and the hole at the center is induced by the hydrogen gas released by the cathode reaction. EDS results (Table 4) reveal that the bulge is Mg(OH)_2_ or MgO due to the high content of Mg and O. Some corrosion products with a high content of Ca/P (Position II) are deposited around the volcano-like bulge, and can be denoted as Ca/Mg-(hydro)phosphate/carbonate. For the as-extruded alloy, the substrate is corroded slightly, but pitting corrosion pits are formed on the surface. The areas marked by arrows in Figure 8d reveal some small pits left by the detachment of the W-phase. Figure 8e shows that the corrosion layer has a local rupture caused by pitting corrosion. Some clustered particles attached to the surface, and EDS results indicate that the clustered particles have high Ca/P content, similar to Position II and Position V, which indicates that these corrosion products are Ca/Mg-(hydro)phosphate/carbonate but with different shapes. The longitudinal section images of the as-cast and as-extruded alloys (Figure 8c,f, respectively) exhibit a two-tier structure, with an inner layer consisting of Mg(OH)_2_ or MgO and the outer layer comprising Ca/Mg-(hydro)phosphate/carbonate.

**Figure 9:**
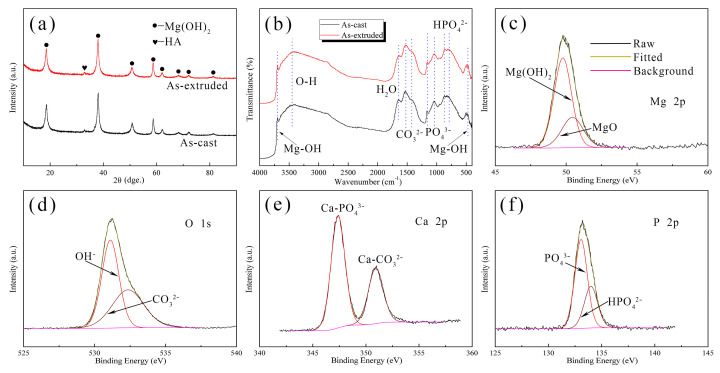
(**a**) XRD patterns, (**b**) FTIR spectra, and (**c**–**f**) XPS spectra with high-resolution scanning of Mg 2p, O 1s, Ca 2p, and P 2p of the corrosion products after immersion in SBF for 240 h.

Figure 9a shows the XRD patterns of the corrosion products after immersion in SBF for 240 h. The corrosion products of the as-cast and as-extruded alloys are mainly composed of Mg(OH)_2_ and a small amount of Ca_10_(PO_4_)_6_(OH)_2_ (hydroxyapatite, i.e., HA). No other compounds are detected due to their low content. FTIR analysis was carried out to further verify the composition of corrosion products (Figure 9b). The broad adsorption peak at 3450 cm^−1^ corresponds to the stretching vibration of O-H [46], and the sharp peaks at 3700 cm^−1^ and 470 cm^−1^ are attributed to the O-H stretching vibration in the crystal structure of Mg(OH)_2_ [47,48]. This finding further proves the presence of Mg(OH)_2_. The absorption peaks at 1522 cm^−1^ and 1422 cm^−1^ arise from the antisymmetric stretching vibrations of CO_3_^2−^ [49]. The absorption band from 1200 to 1000 cm^−1^ is ascribed to PO_4_^3−^ vibration [50,51], and the peaks at 872 cm^−1^ and 788 cm^−1^ correspond to the vibration of HPO_4_^2−^ [49]. The peak at 1646 cm^−1^ corresponds to H_2_O bending vibration, which indicates the existence of crystal water in the corrosion layer [46].

Figure 9c–f show the Mg 2p, O 1s, Ca 2p, and P 2p XPS spectra of the corrosion products after 240 h immersion. The binding energy for Mg 2p is assigned to Mg(OH)_2_ (49.8 eV) and MgO (50.5 eV) [52]. MgO is derived from the decomposition of Mg(OH)_2_, which usually coexists in corrosion products [53]. The O 1s peak at 531.1 eV corresponds to OH^−^, and the other peak at 532.4 eV is attributed to CO_3_^2−^ [54]. Ca 2p is detected as double peaks at 347.4 eV and 351 eV, which correspond to Ca-PO_4_^3−^ and Ca-CO_3_^2−^, respectively [55]. The P 2p peak can be resolved into the PO_4_^3−^ peak at 133.1 eV and the HPO_4_^2−^ peak at 134 eV [56]. Combined with XRD and FTIR results, the corrosion products mainly contain Mg(OH)_2_, MgO, HA, carbonate, phosphate, and hydrophosphate.

### 3.4. In Vitro Cytocompatibility

**Figure 10:**
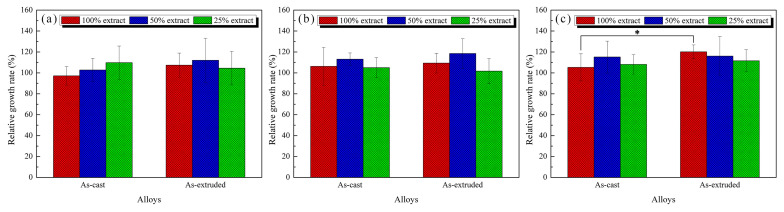
RGRs of L-929 cells after (**a**) 24 h, (**b**) 48 h, and (**c**) 72 h incubation at different extract concentrations. * *p* < 0.05.

Figure 10 shows the relative growth rates (RGRs) of the L-929 cells after 24 h, 48 h, and 72 h incubation. According to ISO 10993-5:2009, cytotoxicity can be divided into Grades 0–5, of which Grades 0 (RGR ≥ 100%) and 1 (75–99%) are considered safe. The RGRs of the L-929 cells cultured at different extract concentrations are all greater than 75%, which indicates the good biosafety of Mg-1.5Zn-1.2Y-0.1Sr alloys. There are no significant differences in RGRs between the as-cast and as-extruded alloys after 24 h and 48 h incubation. However, after 72 h incubation, the RGR of the as-extruded alloy in 100% extracts was considerably higher than that of the as-cast alloy, reaching 120% ± 7%. Such findings may be related to the facilitation effect of released ions during immersion (for instance, Mg^2+^ and Zn^2+^). Similarly, the RGRs of as-cast and as-extruded alloys showed no significant difference for 50% and 25% extracts.

**Figure 11:**
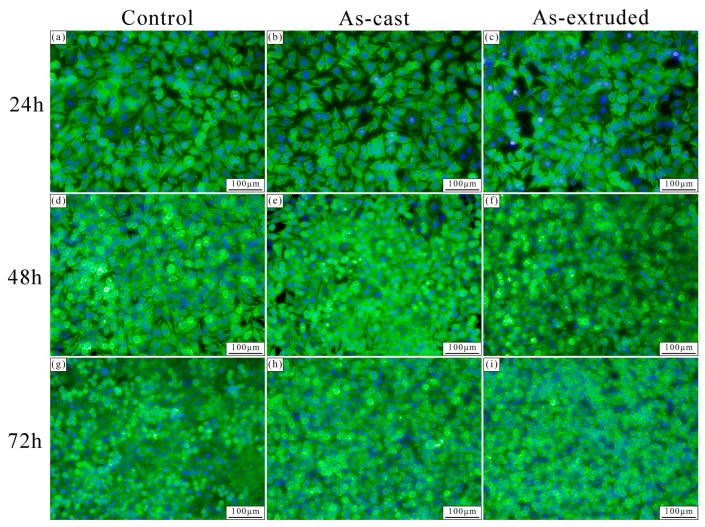
Cell morphology after culturing for (**a**–**c**) 24 h, (**d**–**f**) 48 h, and (**g**–**i**) 72 h in DMEM (control group) and 100% extracts of the as-cast and as-extruded alloys.

Figure 11 shows the cell morphology after culturing in 100% extract for 24 h, 48 h, and 72 h. In the early stages of incubation (24 h), sufficient growth space allows the cell filopodia to gradually extend to the surroundings, which makes them appear to be polygonal and spindle-shaped. With the proliferation of cells (48 h), the bottom of the culture plate is almost completely occupied by cells, and the limited space prevents cells from fully spreading out. Thus, a few round cells are observed (Figure 11d–f). After 72 h of culture, the newly proliferated cells aggregate into clusters, and the crowded living space prevents all cells from expanding outward. Hence, only circular-shaped cells are observed.

**Figure 12:**
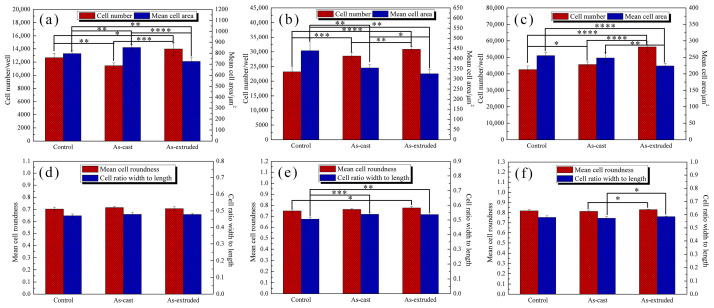
High-content analysis of L-929 cells after culturing for (**a**,**d**) 24 h, (**b**,**e**) 48 h, and (**c**,**f**) 72 h in 100% extracts. * *p* < 0.05, ** *p* < 0.01, *** *p* < 0.001, **** *p* < 0.0001.

To obtain more information about cells, we conducted a quantitative analysis of the cell number, mean cell area, mean cell roundness, and width to length ratio of the L929 cells grown in Mg 100% extract using HCS analysis (Figure 12). The number of cells is proportional to RGRs (Figure 10), and a significant difference among control, as-cast, and as-extruded alloys is identified. This finding demonstrates the limitation of MTT to evaluate the cytotoxicity. The average cell area decreases with the increase in cell number, which is attributed to the limited space during cell proliferation. After 24 h of culture, small cell roundness and width to length ratio are observed due to the fusiform or polygonal morphology. However, with the increase in the round cell number, the roundness and width to length ratio gradually increase, especially after 72 h incubation, where the mean cell roundness reaches 0.8, and the width to length ratio is close to 0.6. The changes in these parameters reflect the survival state of cells during proliferation.

**Figure 13:**
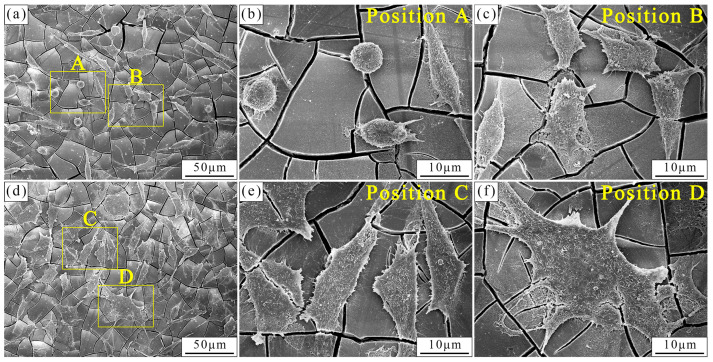
Cell adhesion on the (**a**–**c**) as-cast and (**b**–**f**) as-extruded alloys after culturing for 24 h.

Figure 13 presents the adhesion of L929 cells to the surface of as-cast and as-extruded alloys after 24 h incubation. The cells on the as-cast alloy show spherical and fusiform shapes, which have not spread out sufficiently. Only a few filopodia are observed. By contrast, the cells that attach to the as-extruded alloy surface are polygonal and flatter than those on the as-cast alloy. Numerous filopodia extend from the body and connect to other cells, which indicates that the as-extruded alloy surface provides a favorable site for cell attachment and growth [57].

## 4. Discussion

### 4.1. Evolution of Mechanical Properties of the As-Cast and As-Extruded Mg-1.5Zn-1.2Y-0.1Sr Alloys

**Figure 14:**
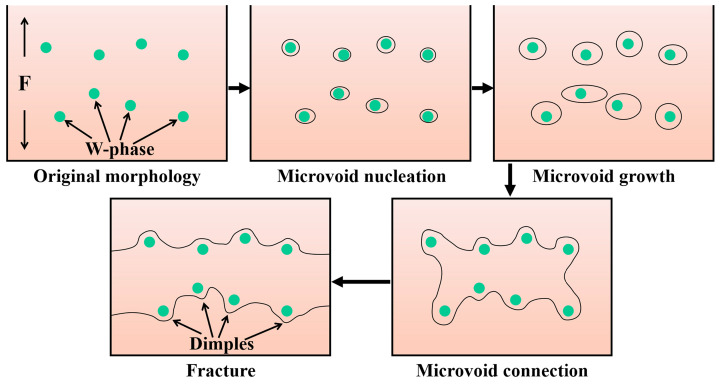
Schematic diagram of microvoid coalescence fracture in the as-extruded alloy.

Different microstructures are the main reason for the variation in mechanical property between the as-cast and as-extruded Mg-1.5Zn-1.2Y-0.1Sr alloys. Figure 1 shows that the grain size of the as-extruded alloy is remarkably smaller than that of the as-cast alloy. According to the Hall–Petch formula, the smaller the grain size, the greater the YS. Thus, the YS of the as-extruded alloy is considerably higher than that of the as-cast alloy. In addition, the distribution and size of the W-phase are crucial factors affecting mechanical properties. In the as-cast alloy, during the tensile process, stress concentration easily occurs near the coarse W-phase and induces the nucleation of microcracks. As the load increases, microcracks propagate along specific crystal planes (e.g., {0001}), and eventually result in transgranular cleavage fracture. Transgranular cleavage fracture is a brittle fracture, and thus the mechanical properties of the as-cast alloy are unsatisfactory. In the as-extruded alloy, the size of the W-phase, which is dispersed within the substrate, decreases substantially, and this condition results in the fracture mode transforming into microvoid coalescence fracture. Figure 14 vividly illustrates the process of the microvoid coalescence fracture, which can be classified into four stages: microvoid nucleation, microvoid growth, microvoid connection, and fracture [25]. At the initial stage of stress, the dispersed particles (broken W-phase) first separate from the α-Mg matrix and form microvoids due to stress concentration. At this point, the microvoids have a very small diameter. However, as the load is continuously applied, the microvoids gradually grow and combine, leading to the formation of wide crack. Eventually, fracture occurs. Given that the nucleation and growth of microvoids are accompanied by a large amount of plastic deformation, the as-extruded alloy often exhibits better mechanical properties than the as-cast alloy. Hence, dispersion strengthening is one of the crucial reasons for the excellent mechanical properties of the as-extruded alloy.

**Figure 15:**
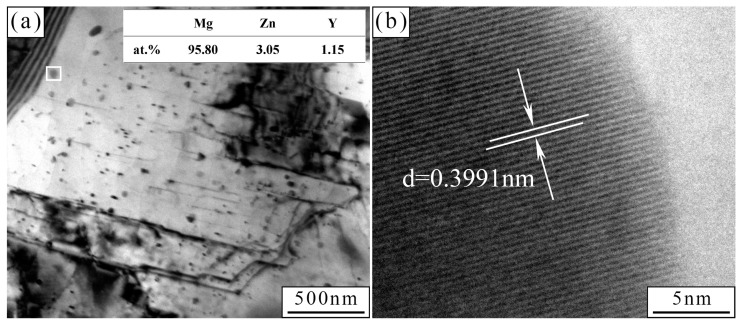
(**a**) Nanoscale precipitates and dislocations in the as-extruded alloy; (**b**) HRTEM of nanoscale precipitates in the as-extruded alloy. The inset in (**a**) represents the EDS results of the white box.

The drastic increase in dislocation density after hot extrusion also results in improved mechanical properties (Figure 15). In the process of dislocation movement, dislocation lines are prone to cross each other, which causes dislocation entanglement and hinders dislocation movement to improve strength. Furthermore, numerous fine particles (EDS result shows that they are W-phase) are precipitated in the grain, and they also become a barrier to dislocation movement. In conclusion, the strengthening mechanism of the as-extruded Mg-1.5Zn-1.2Y-0.1Sr alloy can be attributed to refinement strengthening, dispersion strengthening, dislocation strengthening, and precipitation strengthening.

### 4.2. Corrosion Evolution of the As-Cast and As-Extruded Mg-1.5Zn-1.2Y-0.1Sr Alloys

**Figure 16:**
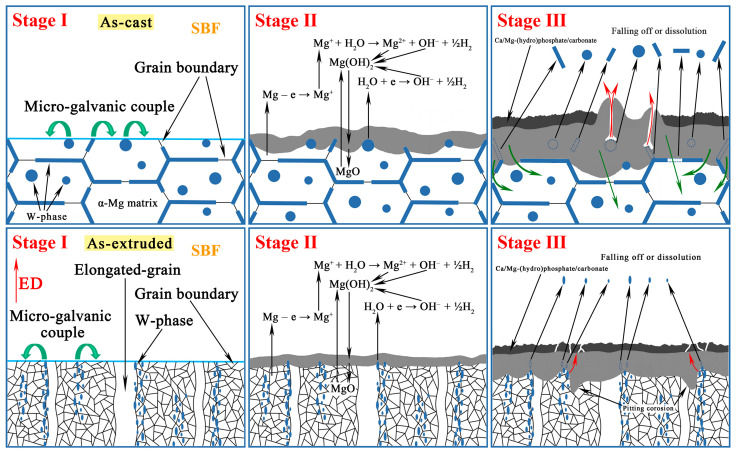
Corrosion mechanism of the as-cast and as-extruded Mg-1.5Zn-1.2Y-0.1Sr alloys.

Figure 8c,f show that galvanic corrosion is dominant in the as-cast and as-extruded alloys, respectively, and the potential of W-phase is higher than that of the α-Mg matrix. We clarified the galvanic corrosion process for the as-cast and as-extruded alloys. The schematic diagram is shown in Figure 16. Stage I represents the original morphology. When the as-cast alloy is in contact with SBF, electrochemical reactions occur immediately (Stage II). The corrosion process can be explained by the following equations [27]:

(2) $$\mathrm{Mg}\;-\;e\;\to \;{\mathrm{Mg}}^{+};\;(\mathrm{anodic}\;\mathrm{reaction})$$

(3) $${\mathrm{Mg}}^{+}+H_{2}O\to {\mathrm{Mg}}^{2+}+{\mathrm{OH}}^{-}+1/2H_{2};\;(\mathrm{chemical}\;\mathrm{reaction})$$

(4) $$H_{2}O+e\;\to \;{\mathrm{OH}}^{-}+1/2H_{2};\;\left(\mathrm{cathodic}\;\mathrm{reaction}\right)$$

(5) $$\mathrm{Mg}+2H_{2}O\;\to \;{\mathrm{Mg}}^{2+}+2{\mathrm{OH}}^{-}+H_{2};\;(\mathrm{overall}\;\mathrm{reaction})$$

(6) $${\mathrm{Mg}}^{2+}+2{\mathrm{OH}}^{-}\to {\;\mathrm{Mg}(\mathrm{OH})}_{2}.\;(\mathrm{product}\;\mathrm{formation})$$

Initially, the α-Mg matrix is dissolved and Mg^+^ is produced at the anode site. The intermediate Mg^+^ is unstable and reacts with H_2_O to form Mg^2+^ and H_2_. Meanwhile, the cathodic reaction also produces H_2_. Hence, gas is visible when the sample is immersed in the SBF. In this process, the pH of the electrolyte increases due to the generation of OH^−^, which can be reflected in Figure 6a. The high concentrations of Mg^2+^ and OH^−^ on the surface combine with each other to form Mg(OH)_2_. Meanwhile, the part of Mg(OH)_2_ near the substrate dehydrates to form MgO. This process is reversible. As the alloy corrosion becomes increasingly serious, the W-phase falls off or dissolves, and the electrolyte penetrates the interior of the alloy (Stage III). Hence, huge corrosion pits are observed in the as-cast alloy (Figure 8a). Subsequently, the Ca/Mg-(hydro)phosphate/carbonate is deposited on the surface of Mg(OH)_2_. According to the XRD, FTIR, and XPS results, the formation of Ca/Mg-(hydro)phosphate/carbonate mainly involves the following reactions:

(7) $${{\mathrm{HCO}}_{3}}^{-}+{\mathrm{OH}}^{-}\to {{\mathrm{CO}}_{3}}^{2-}+H_{2}O;$$

(8) $${H_{2}{\mathrm{PO}}_{4}}^{-}+{\mathrm{OH}}^{-}\to {{\mathrm{HPO}}_{4}}^{2-}+H_{2}O;$$

(9) $${{\mathrm{HPO}}_{4}}^{2-}+{\mathrm{OH}}^{-}\to {{\mathrm{PO}}_{4}}^{3-}+H_{2}O;$$

(10) $${\mathrm{Ca}}^{2+}+{{\mathrm{CO}}_{3}}^{2-}\to {\mathrm{CaCO}}_{3}\;\left(s\right);$$

(11) $${3\mathrm{Ca}}^{2+}+2{{\mathrm{PO}}_{4}}^{3-}\to \;{\left(\mathrm{Ca}\right)}_{3}{{(\mathrm{PO}}_{4})}_{2}\left(s\right);$$

(12) $${\mathrm{Mg}}^{2+}+{\mathrm{Ca}}^{2+}+{{\mathrm{HPO}}_{4}}^{2-}\to \;(\mathrm{Mg},\;\mathrm{Ca}){\mathrm{HPO}}_{4}\;\left(s\right);$$

(13) $${10\mathrm{Ca}}^{2+}+6{{\mathrm{PO}}_{4}}^{3-}+2{\mathrm{OH}}^{-}\to \;{\mathrm{Ca}}_{10}{\left({\mathrm{PO}}_{4}\right)}_{6}{\left(\mathrm{OH}\right)}_{2}(s).\;$$

Hence, the Ca/Mg-(hydro)phosphate/carbonate is mainly composed of HA, CaCO_3_, (Ca)_3_(PO_4_)_2_, and hydrophosphate. Hydrophosphate is possibly (Mg, Ca)HPO_4_. The Ca/Mg-(hydro)phosphate/carbonate layer formed on the surface can alleviate the penetration of the electrolyte and slow down the corrosion rate. However, given the accumulation of Mg(OH)_2_, a volcanic-like morphology is formed which results in the destruction of the Ca/Mg-(hydro)phosphate/carbonate that accelerates the corrosion rate. This finding also explains the poor corrosion resistance of the as-cast alloy.

The corrosion mechanism of the as-extruded alloy is similar to that of the as-cast alloy, and the intensity of galvanic corrosion is considerably weakened because of the reduced size and volume fraction of the W-phase. In the early stage of corrosion, the substrate is slightly corroded, and only a thin layer of Mg(OH)_2_ along with a small amount of MgO is formed on the surface (Stage II). However, with the advancement of immersion time, pitting corrosion occurs around the W-phase and develops inward along the distribution of the W-phase (Stage III). Hence, pitting corrosion pits can be observed in Figure 8d. Lastly, like the as-cast alloy, the Ca/Mg-(hydro)phosphate/carbonate is deposited on the Mg(OH)_2_ surface, but the Ca/Mg-(hydro)phosphate/carbonate layer cracks in the area with severe pitting corrosion (Figure 8e), which is unfavorable to the alloy.

From the above discussion, the size and distribution of the W-phase were the most important factors that affect corrosion resistance. However, other factors cannot be ignored. On the one hand, the process of extrusion can eliminate casting defects, such as shrinkage, which is one of the reasons for the good corrosion resistance of the as-extruded alloy. On the other hand, grain refinement promotes the improvement of corrosion resistance. Previous reports proved that grain refinement contributes to a protective oxidation film formed on the surface [58], and grain boundaries can act as a physical barrier to prevent the spread of corrosion [59]. Hence, these factors render the as-extruded alloy with a good corrosion resistance.

### 4.3. Evolution of In Vitro Biocompatibility of the As-Cast and As-Extruded Mg-1.5Zn-1.2Y-0.1Sr Alloys

The biocompatibility of Mg-1.5Zn-1.2Y-0.1Sr alloys is investigated via indirect (MTT and HCS analysis) and direct (cell adhesion) methods. Although cytotoxicity did not show a significant difference until 72 h incubation, HCS analysis exhibited a significant difference in cell parameters (for example, cell number). Therefore, it is necessary to conduct an in-depth investigation of the influencing factors of cytotoxicity. The following are discussed from the aspects of pH, ion concentration, and hydrogen evolution.

**Figure 17:**
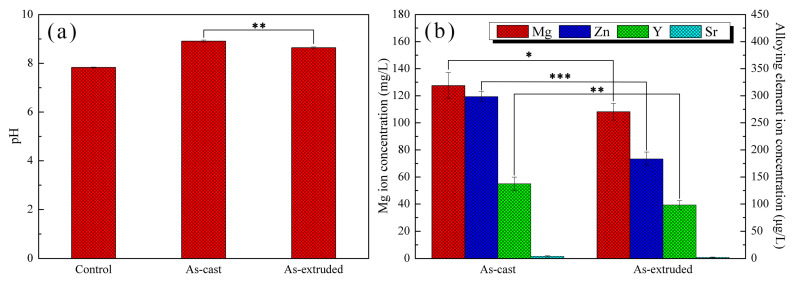
(**a**) pH and (**b**) ion concentrations of the extracts after 72 h immersion. * *p* < 0.05, ** *p* < 0.01, *** *p* < 0.001.

The pH of extracts is an important factor affecting cell viability. The suitable pH range for cell growth is 7.4–7.8, and an extremely high or extremely low pH will lead to the inhibition of protein synthesis [60]. Figure 17a shows that the pH of 100% extract of the as-cast alloy was considerably higher than that of the as-extruded alloy after the immersion in DMEM for 72 h. Therefore, the cell number in the 100% extract of the as-cast alloy was consistently smaller than that in the 100% extract of as-extruded alloy. This outcome proves that a high pH is detrimental to cell proliferation. However, after incubation for 48 h and 72 h, the cell number in the 100% extract of the as-cast alloy exceeded that in the control group, which indicates that the cells gradually adapt to the high-pH environment. The cells show similar RGRs in different extracts, which manifests the limited influence of pH. Other factors cause the as-cast and as-extruded alloys to exhibit different cell viability results.

The Mg ion and alloying element ions released during the degradation of the alloy in DMEM also affect cell viability. Figure 17b shows the ion concentrations of the as-cast and as-extruded alloys after 72 h immersion in DMEM. The concentration of Mg ions is the highest and has the greatest effect on cell viability. Zhen et al. [61] showed that the viability of L929 cells decreased sharply when the Mg ion concentration exceeded 100 mg/L because high concentrations of Mg ions can influence the osmotic pressure of the medium and the biological effect of Ca ions that play a crucial role in metabolism. Hence, the high concentrations of Mg ions explain why the cell number in the 100% extract of the as-cast alloy is inferior to that of the as-extruded alloy. The concentration of Zn ion is second to that of Mg ions, but the content is notably lower than that of Mg ions. As reported by G. Schmalz et al. [62], the IC_50_ of Zn ions for L929 is 25.5 μM, which is substantially higher than that of Zn ions in our test; thus, Zn does not have a negative effect on cell proliferation. By contrast, Gu et al. [12] observed that the Mg-1Zn alloy extract showed an increased cell viability towards L929 cells at the Zn ion concentration of 2.6 ± 1 μM. The high RGRs in the 100% extract of the as-extruded alloy can be related to the promotion of Zn ions. As for Y, A. Yamamoto et al. [63] showed that the IC_50_ of Y ions for L929 was 2.54 × 10^−4^ M. Thus, the concentration of Y ions in our experiment is safe. However, Grade 1 toxicity was observed when the Y ion concentration was 2.3 ± 0.7 μM, which indicates that the high Y content is one of the reasons for the poor cell viability of the as-cast alloy after 24 h incubation in 100% extract. The content of Sr in the medium is extremely low, and its effect on cells is limited, and thus will not be discussed in detail.

Hydrogen evolution mainly affects cell adhesion to the alloy surface. When the alloy is in contact with DMEM, the hydrogen evolution reaction occurs. The outward diffusion of hydrogen influences the migration and growth of cells on the alloy surface. Given the existence of the coarse W-phase in the as-cast alloy, the hydrogen evolution reaction is extremely severe. As a result, most of the cells attached to the as-cast alloy surface have a spherical and fusiform shape (Figure 13a). Meanwhile, the local high pH on the as-cast surface caused by hydrogen evolution can lead to cell death. By contrast, a majority of cells on the as-extruded alloy surface appear polygonal and more fully spread out, which indicates that hydrogen evolution has minimal effect on cell adhesion. In other words, the slight surface reaction of the as-extruded alloy is conducive to cell adhesion.

## 5. Conclusions

In this work, a novel alloy with Mg-1.5Zn-1.2Y-0.1Sr (wt.%) composition was designed for biomedical biodegradable implants. The corrosion behavior, fracture mechanism, and in vitro biocompatibility were discussed in detail. The main conclusions are as follows.

(1) The Mg-1.5Zn-1.2Y-0.1Sr alloys were mainly composed of an α-Mg matrix and W-phase. The W-phase in the as-cast alloy was primarily distributed at the grain boundaries, and a small amount of granular W-phase was precipitated in the grains. After hot extrusion, the size of the grains and the W-phase decreased considerably. The as-extruded alloy exhibited a multiscale structure, which can be classified as micron/nanosized precipitates and fine, coarse, and elongated grains.

(2) The as-cast alloy was dominated by transgranular cleavage rupture with a poor tensile property. After hot extrusion, the fracture mode changed to microvoid coalescence fracture. The as-extruded alloy presented appropriate mechanical properties, with UTS, YS, and EL values of 279.1 ± 2.4 MPa, 234.3 ± 7.4 Mpa, and 28.7% ± 1.1%, respectively. Refinement strengthening, dispersion strengthening, dislocation strengthening, and precipitation strengthening were the main strengthening mechanisms in the as-extruded alloy.

(3) The main corrosion mechanism in the as-cast and as-extruded alloys was the galvanic corrosion between the W-phase and the α-Mg matrix. The as-extruded alloy exhibited a higher corrosion resistance than the as-cast alloy. The coarse W-phase was the direct reason for the high corrosion rate of the as-cast alloy. The broken W-phase in the as-extruded alloy weakened the galvanic corrosion intensity, but pitting corrosion was observed. After 240 h of immersion in SBF, the corrosion rate of the as-extruded alloy was lower than 0.5 mm/year. The corrosion products that formed on the as-cast and as-extruded alloy surface mainly contained Mg(OH)_2_, MgO, HA, CaCO_3_, (Ca)_3_(PO_4_)_2_, and hydrophosphate. Hydrophosphate was possibly (Mg, Ca)HPO_4_.

(4) The pH, ion concentration, and hydrogen evolution were the main factors affecting in vitro biocompatibility. HCS provided richer information than MTT in the evaluation of cytotoxicity. MTT, HCS analysis, and cell adhesion showed that the as-extruded alloy can promote L929 cell growth and proliferation.

(5) The as-extruded Mg-1.5Zn-1.2Y-0.1Sr alloy showed high suitability for use in implant materials. However, pitting corrosion limits its application, and mechanical properties have the potential to be improved. These problems need to be studied further.

## Figures and Tables

**Table 1:** Chemical composition of the as-cast Mg-1.5Zn-1.2Y-0.1Sr (wt.%) alloy.

Zn	Y	Sr	Fe	Si	Mn	Al	Mg
1.4724	1.1923	0.1028	0.0036	0.0027	0.0018	0.0009	Bal.

**Table 2:** Corrosion potential (E_corr_), corrosion current density (J_corr_), and pitting potential (E_pt_) obtained from PDP curves.

Alloys	E_corr_ (V)	J_corr_ (μA/cm^2^)	E_pt_ (V)
As-cast	−1.625 ± 0.007	45.548 ± 3.191	−1.510 ± 0.009
As-extruded	−1.584 ± 0.012	17.492 ± 1.570	−1.549 ± 0.011

**Table 3:** Fitting results obtained from EIS data.

Samples	R_s_ (Ωcm^2^)	CPE_f_ (s^n^Ω^−1^cm^−2^)	Rf (Ωcm^2^)	n_1_	CPE_dl_ (s^n^Ω^−1^cm^−2^)	R_ct_ (Ωcm^2^)	n_2_	R_L_ (Ωcm^2^)	L (Hcm^2^)
As-cast	7.331	1.87 × 10^−5^	85.07	0.72	1.37 × 10^−5^	5993	0.86	9067	755,160
As-extruded	13.990	2.58 × 10^−5^	306.6	0.65	5.00 × 10^−6^	9218	0.94	26,266	915,480

**Table 4:** Chemical composition (at.%) of the different areas marked in Figure 8.

Positions	C	O	Mg	P	Ca	Na	Cl
I	8.91	69.00	21.81	0.09	-	0.19	-
II	6.71	69.52	13.87	5.26	4.25	-	0.39
III	6.90	67.58	7.36	8.56	9.19	0.41	-
IV	7.54	67.43	24.39	0.48	0.16	-	-
V	6.15	61.24	15.13	8.00	8.80	0.53	0.15
VI	7.29	65.99	7.03	9.00	10.23	0.46	-
VII	5.85	60.63	15.76	7.97	9.27	0.52	-
VIII	8.62	67.74	22.57	0.49	0.58	-	-

## Data Availability

The raw/processed data required to reproduce these findings cannot be shared at this time as the data also form part of an ongoing study.
